# A retrospective chart review to identify perinatal factors associated with food allergies

**DOI:** 10.1186/1475-2891-11-87

**Published:** 2012-10-19

**Authors:** Kelly Dowhower Karpa, Ian M Paul, J Alexander Leckie, Sharon Shung, Nurgul Carkaci-Salli, Kent E Vrana, David Mauger, Tracy Fausnight, Jennifer Poger

**Affiliations:** 1 Pennsylvania State University College of Medicine, 500 University Drive, Hershey, PA, 17033, USA

**Keywords:** Antibiotics, Atopic dermatitis, Bifidobacteria, Cesarean section, Food allergy, Group B *Streptococcus*, Gut flora, *Lactobacillus*, PBMC peripheral blood mononuclear cell

## Abstract

**Background:**

Gut flora are important immunomodulators that may be disrupted in individuals with atopic conditions. Probiotic bacteria have been suggested as therapeutic modalities to mitigate or prevent food allergic manifestations. We wished to investigate whether perinatal factors known to disrupt gut flora increase the risk of IgE-mediated food allergies.

**Methods:**

Birth records obtained from 192 healthy children and 99 children diagnosed with food allergies were reviewed retrospectively. Data pertaining to delivery method, perinatal antibiotic exposure, neonatal nursery environment, and maternal variables were recorded. Logistic regression analysis was used to assess the association between variables of interest and subsequent food allergy diagnosis.

**Results:**

Retrospective investigation did not find perinatal antibiotics, NICU admission, or cesarean section to be associated with increased risk of food allergy diagnosis. However, associations between food allergy diagnosis and male gender (66 vs. 33; p=0.02) were apparent in this cohort. Additionally, increasing maternal age at delivery was significantly associated with food allergy diagnosis during childhood (OR, 1.05; 95% CI, 1.017 to 1.105; p=0.005).

**Conclusions:**

Gut flora are potent immunomodulators, but their overall contribution to immune maturation remains to be elucidated. Additional understanding of the interplay between immunologic, genetic, and environmental factors underlying food allergy development need to be clarified before probiotic therapeutic interventions can routinely be recommended for prevention or mitigation of food allergies. Such interventions may be well-suited in male infants and in infants born to older mothers.

## Background

**Figure 1:**
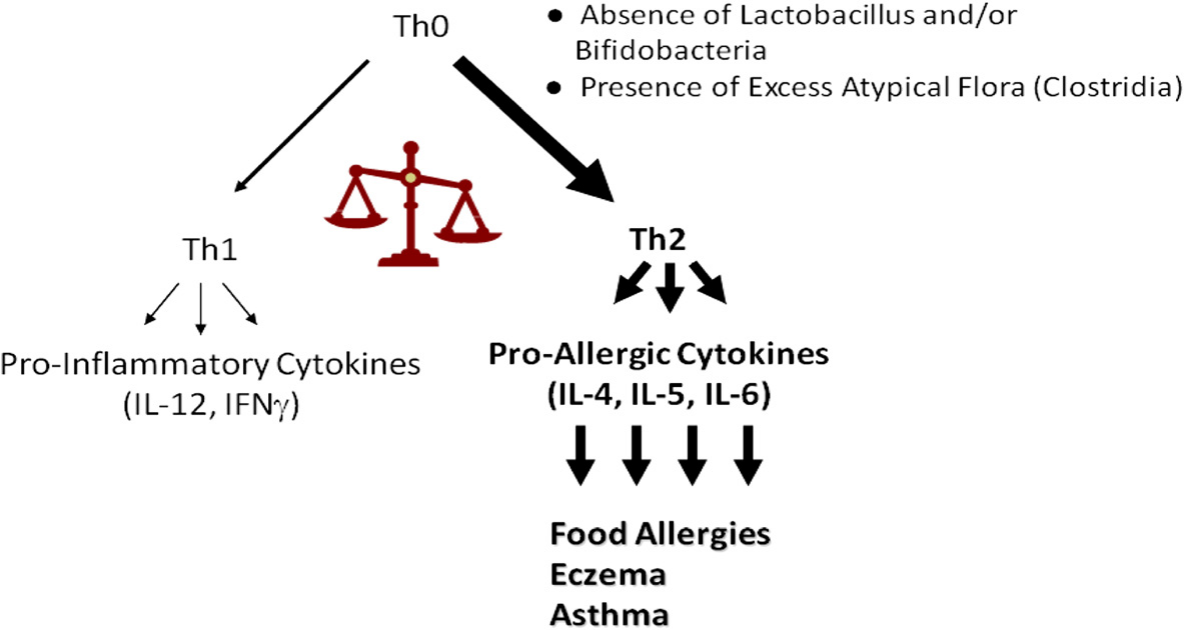
**Depiction of gut flora contribution to atopy**. The neonatal immune system (Th0) is predisposed to develop Th2 responses, especially in the presence of atypical gut flora or the absence of lactobacilli and/or bifidobacteria. Therapies that stimulate Th1 responses may be able to restore balance and lead to immunologic tolerance rather than hypersensitivity.

Distinct differences in intestinal microbiota have been identified in children with atopic conditions compared to their non-allergic peers [1]. Specifically, current evidence suggests that decreased numbers of lactic acid-producing bacteria and/or increased numbers of pro-inflammatory microorganisms in the gastrointestinal tract of infants may predispose to atopic dermatitis. During the first year of life, fewer gastrointestinal bifidobacteria (and concomitantly more clostridia and *Staphylococcus aureus*) have been identified in children who are diagnosed with atopic conditions [2]. Similarly, in rodent models of atopy, *Bifidobacterium* and/or *Lactobacillus sp*. have been found to repress cellular and humoral responsiveness in milk-allergic mice and even restore oral tolerance [3,4]. Taken together, these pre-clinical and clinical observations suggest that a critical balance of gut flora is needed for oral tolerance and appropriate immune maturation such that specific atopic manifestations can be avoided [5,6] (Figure 1).

Probiotics are live microorganisms that provide health benefits when ingested in adequate quantities. These bacteriotherapies are increasingly used by consumers and recommended by health care providers including pharmacists and nutritionists [7-9]. Probiotics, used as dietary supplements, have been investigated as a means of preventing development of childhood atopic conditions, albeit with conflicting results [10-15].

Preliminary work from our laboratory suggests that co-incubation of probiotic bacteria with peripheral blood mononuclear cells (PBMCs) induces cytokine responses in a manner that is consistent with responses observed in vivo during induction of oral tolerance [16-21]. Indeed, we find that PBMCs respond to probiotics with a heightened release of IFN-γ, IL-10, and IL-12; furthermore, some of these effects are observed to be more robust in cells obtained from cord blood than from adult donor cells (Additional file 1). These results suggest that appropriately-selected and suitably-dosed probiotic supplements have potential to prevent/restore aberrant Th2 responses by shifting immunity in favor of a Th1-type phenotype.

If appropriately selected bacterial therapies can positively impact oral tolerance mechanisms, it is likely that these dietary supplements would mediate their greatest effects in individuals with disrupted intestinal flora. Factors known to disrupt neonatal acquisition of gut flora include cesarean section delivery, use of antibiotics, and time spent in a neonatal intensive care unit (NICU) [22-34]. We hypothesized that factors known to disturb perinatal acquisition of gut flora would increase the risk of subsequent food allergy diagnosis in childhood. Such a correlation would be useful in identifying children at greatest risk of food allergies such that appropriate immunomodulatory interventions could be implemented. Our hypothesis was tested via a retrospective chart review of birth records. We identified gender in children and age of mother at time of delivery as being associated with increased risk of food allergies in children, but did not find associations between food allergies and antibiotic exposure, delivery method, or neonatal nursery.

## Methods

### Retrospective chart review inclusion criteria

With approval and oversight from the Institutional Review Board at Penn State Hershey Medical Center (PSHMC) (IRB # 24958EP), a retrospective chart review was undertaken to identify children visiting either (a) a PSHMC primary care practice (controls) or (b) allergy specialty clinic for a food allergy-related concern (cases) who were also born at the institution’s medical center. Penn State Hershey Children’s Hospital has more than a million children in the referral area and offers the only Level I pediatric trauma center in more than 70,000 square miles. Similarly, the allergy clinic is a physician-referral clinic that sees complex patients; the catchment area for the allergy clinic covers more than 85,000 square miles of central and eastern Pennsylvania and accepts both private and public insurances.

To identify allergic children, the ICD-9-CM coding system was used to identify clinic visits that were coded in a manner consistent with food-related allergic reactions (Table 1). Those codes were subsequently matched to children who were born at PSHMC between July 1, 2000, and June 30, 2005. The children identified as a result of this match were considered to be “potential cases”, and their medical charts were retrieved for review. However, only children whose medical charts confirmed the presence of food allergies, documented by either a positive serum specific IgE test, (specific IgE antibodies >0.35 kU/L) or positive skin prick test (SPT) (≥ 3 mm compared with negative control), were included in our data analysis as allergic “cases”. Children were excluded from our dataset if miscoding errors were identified (e.g., latex allergies instead of food allergies) or if parent reported “food allergy” that was not confirmed by laboratory testing (e.g., parent thinks child is “allergic” to milk because s/he experiences abdominal discomfort). In an attempt to increase our number of allergic cases, a protocol modification was made near the conclusion of our chart review period that also permitted us to accept direct referrals from pediatricians familiar with our study objectives if they were aware of patients with food allergies that had not been identified via billing records (e.g., possibly missed by initial search of billing records due to ICD-9 mis-coding).

**Table 1:** **ICD**-**9 codes used to identify children with food allergies**

**ICD9 Code**	**Diagnosis**
558.3	Allergic gastroenteritis and colitis
693.1	Dermatitis due to food
708.9	Urticaria not otherwise specified
995.6 series	Anaphylactic shock due to food
995.7	Other adverse food reactions, not specified elsewhere
V15.01	Allergy to peanut
V15.02	Allergy to milk
V15.03	Allergy to egg
V15.04	Allergy to seafood
V15.05	Allergy to food additives or other nuts

### Chart review data collection

Age- and sex-matched controls for this analysis included children born at PSHMC, followed by our pediatric clinics for well-visit check-ups, but without evidence of food-mediated reactions; this cohort of children was also identified from billing records. For both control children and case children, data abstracted from birth records of the child and labor/delivery records of the mother were used to investigate perinatal factors known to impact gut flora acquisition. Data retrieved from charts included: delivery type, time spent in the neonatal intensive care unit, evidence of atopy for child and mother (including anaphylaxis, atopic dermatitis, hives, respiratory allergies), maternal group B *Streptococcus* status, maternal receipt of antibiotics during delivery, maternal intent to breastfeed, and age and parity of birth mother.

### Statistical analysis

Chart review analyses were performed using a matched case–control design where nearly every case was matched with 2 controls. A sample size of 200 cases and 400 controls was planned. It was expected that such a sample would provide >90% power to detect differences between cohorts if the odds ratio was 1.8 or higher for variables such as intrapartum antibiotic exposure where use in the control population approximates 30%. Chi square and Fisher exact tests were used for data analysis using SAS version 9.2 (SAS Inc, Cary, NC). Descriptive statistics, including mean, median, standard deviation and quartiles for continuous measures, and frequencies for categorical measures were used to characterize the case and control populations. Logistic regression analysis was used to assess associations between variables of primary interest and food allergy. Strength of associations is expressed using odds ratios with 95% confidence intervals.

## Results

### Neonatal and birth mother retrospective chart review

**Figure 2:**
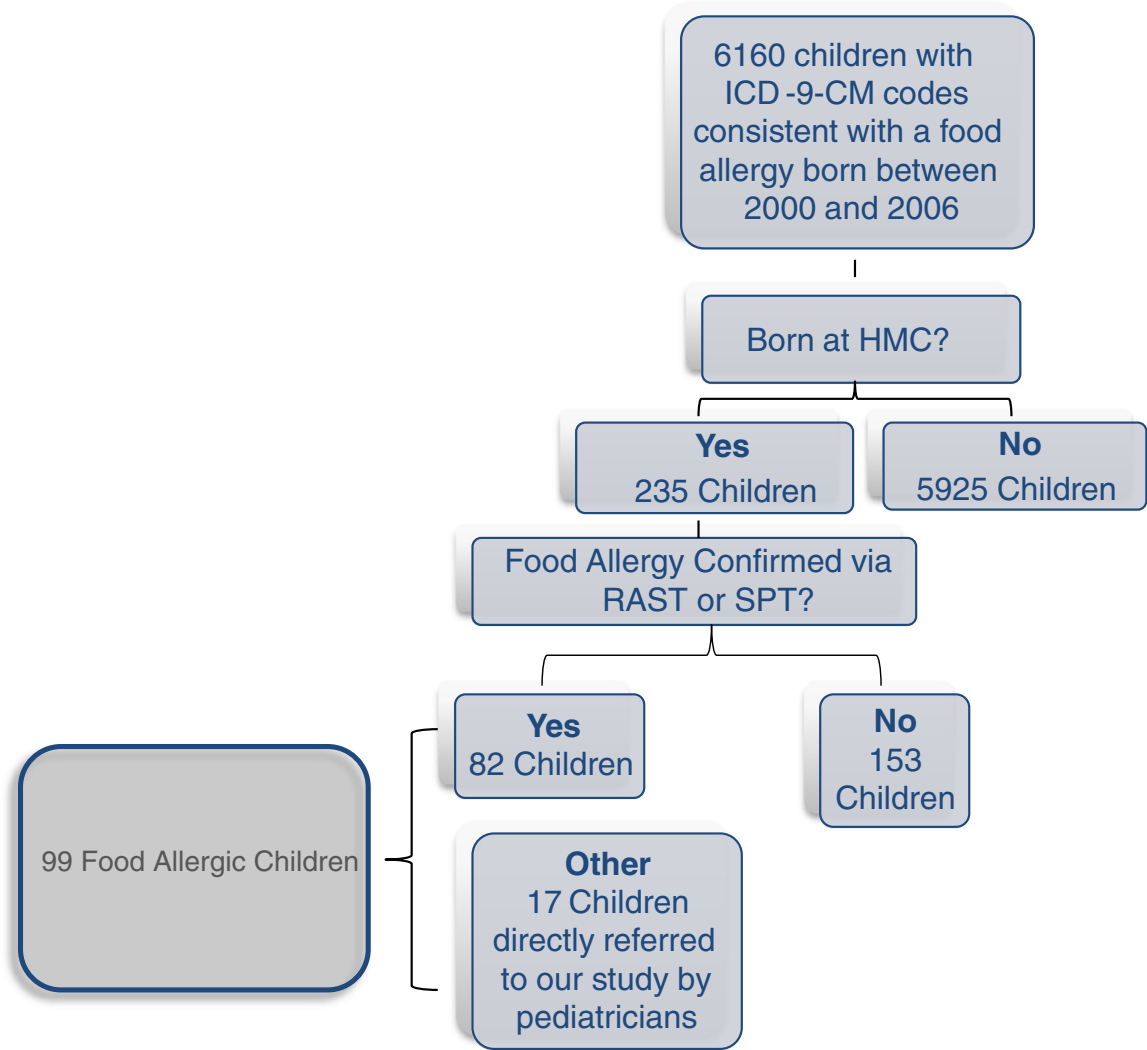
**Identification of Allergic Cohort.** HMC, Hershey Medical Center; RAST, radioallergosorbent test; SPT, skin prick test.

Initial billing records identified 6160 children with ICD-9-CM codes consistent with medical visits related to food allergic reactions between 2000 and 2005 (Figure 2). However, only 235 (3.8%) of those children were born at PSHMC, to permit direct examination of birth records. Of those 235 children (n=125 for males; n=110 for females), a thorough review of medical records confirmed food allergies (positive IgE or SPT) in only 82 (35%) children. In an effort to increase the number of food allergic cases, direct referrals from pediatricians were subsequently accepted; as a result, we expanded the age of children who could be considered as “cases” to 18 years of age based on these direct physician referrals. This protocol modification increased the number of food allergic children (by 17) for whom we could evaluate birth records to 99; three of these children were born in 1998 or 1999. Medical records from 192 non-food allergic children born at the same hospital were also examined as controls. Overall, the mean age of children at the time that charts were reviewed was 5.0 ± 1.5 years, with a mean age of 4.8 ± 1.9 years in the allergic cohort and 5.1 ± 1.3 years (p=0.12) in the non-allergic children.

Of the 291 children (99 cases; 192 controls) included in our analysis, 167 were male and 124 were female (Table 2). There was no difference in gestational age for children with allergies versus non-allergic children (38.3 weeks ± 2.39 weeks; range 27 to 41 weeks versus 38.0 weeks ± 2.86 weeks; range 25 to 42 weeks). There was also no difference in birth weight between the two groups (3.36 kg ± 0.67 kg; range 0.81 to 4.61 kg versus 3.24 kg ± 0.76 kg, range 0.66 to 5.07 kg).

**Table 2:** **Characteristics of food allergic and non**-**allergic children whose birth records were retrospectively reviewed**

	**Allergic**	**Non**-**Allergic**	**P Value**
	**n=99**	**n=192**	
**Gender (n)**			0.02
Males	66	101	
Females	33	91	
**Race/Ethnicity (n)**			0.10
White	79	167	
Black	3	9	
Asian	8	5	
Hispanic	5	5	
Not identified	4	6	
**Parity (n)**			0.93
Zero (excluding this child)	79	154	
≥1 (excluding this child)	20	38	
**Maternal History of Atopy (%)**			0.15
Yes	43	57	
No	32	68	
**Gestational age** (weeks)	38.3	38.0	0.33
**Birth weight** (kg)	3.36	3.24	0.17
**Delivery Type (%)**			0.79
Vaginal	67.7	66.1	
C-section	32.2	33.9	
**Peripartum antibiotics (%)**	28.3	28.1	1.0
**Neonatal antibiotics (%)**	16.2	12.5	0.39
**NICU stay (%)**	17.2	16.7	0.91
**Known positive intent to breastfeed (%)**	88.9	74.2	<0.005
**Known Group B*Streptococcus***			0.72
Positive (%)	18	16	
Negative (%)	32	33	

**Figure 3:**
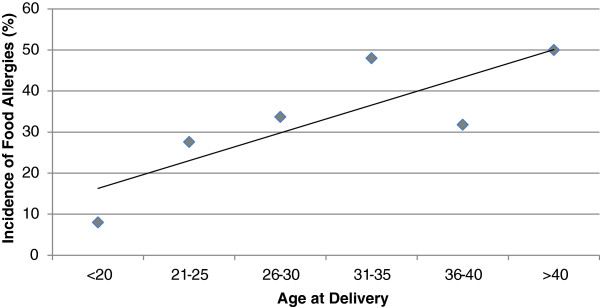
**Incidence of food allergies as a function of maternal age at delivery (years).** Correlation of food allergy diagnosis in childhood and maternal age at delivery. For each advancing year of maternal age, the odds of food allergies diagnosed in newborn increases by 6.0%.

Ethnicity data were available for 282 mothers: 87.6% of mothers self-identified as white (non-Hispanic), whereas the remaining women identified as African American (4.3%), Asian (4.6%) or Hispanic (3.5%). For 45% of the mothers, the children represented by these data were firstborn. In all, 14% of mothers reported a personal history of asthma and 4% of mothers reported food allergies. The likelihood of having a child diagnosed with food allergies was slightly higher for women with a history of atopy compared to women that did not report atopic conditions (43% versus 32%), but this did not reach significance (OR, 1.60; 95% CI, 0.861-2.996; p=0.14). Maternal parity was not associated with an increased incidence of food allergies in offspring, neither did mean maternal age at time of delivery differ significantly between mothers whose children developed food allergies (30.68 ±5.06 years) compared to those that did not (28.55 ± 6.48 years). However, an association was identified between the odds of food allergy diagnosis in childhood and maternal age at delivery (OR =1.05; 95% CI, 1.017 to 1.105; p=0.005). For each advancing year of maternal age at time of delivery, the odds of food allergies being diagnosed in the child increased by 6.0%. This relationship was linear for mothers whose age ranged from under 20 years through 35 years of age (Figure 3).

In the cohort of 99 children with allergies, the mean age at the time of allergy diagnosis was 1.5 years of age and males were significantly more likely to be diagnosed with a food allergy than females (OR = 1.80; 95% CI, 1.088-2.985; p=0.02). Allergies to peanuts, eggs, milk, and other nuts were the most common, impacting 53%, 49%, 43%, and 18% of the food allergic children, respectively. Forty-five percent of children with food allergies were allergic to just one food item, with the remaining children allergic to two or more foods. When these children were exposed to allergenic food(s), they most often experienced skin eruptions, including dermatitis and hives (69%). Anaphylaxis was uncommon (n=10; 5.2%).

No differences were observed with respect to development of food allergies and method of delivery (allergy diagnosis in 34% delivered vaginally and 33% delivered via cesarean; OR=0.93; 95% CI, 0.557-1.564; p=0.79), postnatal antibiotics (40% who developed allergies received antibiotics versus 33% who did not receive antimicrobials; OR=1.35; 95% CI, 0.680-2.677; p=0.39), intrapartum maternal antibiotic exposure (allergy diagnosis in 34% of children whose mothers did or did not receive intrapartum antibiotics; OR=1.00; 95% CI, 0.587-1.715; p=0.998), or time spent in the NICU (allergy diagnosis in 34% and 35% of children who did and did not immediately move to the well child nursery; OR=1.04; 95% CI, 0.544-1.977; p=0.91). Vaginal group B *Streptococcus* (GBS) status was unknown in half the women; for those mothers in whom GBS status was known to be positive, an increased likelihood of developing food allergies was not observed (OR=1.14; 95% CI, 0.556-2.348; p=0.72). Most women (79%) expressed intent to breastfeed their infants; however, intent to breastfeed did not vary between mothers with a positive or negative personal history of atopy (84% versus 78%; p=0.21). A positive correlation between maternal intent to breastfeed and subsequent food allergy development was observed (p<0.005) (Table 2).

## Discussion

### Potential therapeutic implications

Previously, associations between disrupted gut flora and atopic dermatitis have been identified [11,12,35-37]. Furthermore, physiologic evidence links atopic dermatitis with food allergies [38]. Therefore, we wished to determine if an association exists between factors that disrupt perinatal gut flora acquisition and subsequent food allergy diagnosis.

We hypothesized that specific factors known to cause gastrointestinal dysbiosis in newborns -- namely perinatal antibiotic exposure, cesarean section delivery, and NICU admission -- are associated with subsequent food hyper-responsiveness. However, we did not find such correlations to be present in children with confirmed evidence of IgE-mediated food allergies.

In our cohort, we did find, however, that increasing maternal age at delivery is associated with food allergy diagnosis in children. A similar relationship was recently reported by Metsala and colleagues [39]. Although the biologic mechanisms responsible for this association are unclear, it is possible that disrupted maternal normal flora may be involved. It is known that changes in normal flora occur across the lifespan from medications or changes in gastrointestinal tract function. Specifically, with advancing age, levels of gut bifidobacteria and lactobacilli decrease, whereas clostridia and yeast increase [40]. These same changes in gut flora have been previously noted in children with atopy [2]. Thus, while it has not yet been studied, there may be a critical time point during adult life in which mothers may begin to develop disruptions in gut flora, which may then be transferred to children during the delivery process.

We also observed that the number of allergic males in our cohort significantly exceeded the number of allergic females. We are not alone in identifying these gender disparities in atopic sensitization of children. Males have previously been found to have food allergies and other atopic diseases with a higher prevalence than females during childhood and early adolescence [41-46]. Certainly, there may be genetic causes underlying this predisposition, but environmental factors may also play a role. It has been suggested that gender differences in atopic development may reflect a tendency for mothers to breastfeed male infants for a shorter period of time due to the misconception that male infants require a greater level of nutrition than can be provided by breast milk alone [47-50]. Breastfeeding has been reported to provide protection against atopic dermatitis and/or food allergies [51-54]. In addition to nutritive and immunological benefits, breast milk is also known to stimulate growth of bifidobacterial populations. Thus, it is conceivable that breastfeeding duration may play a role in gut flora acquisition and subsequent atopic development. In support of this theory, there is evidence that male infants are indeed more likely than female infants to experience benefits of early probiotic interventions [55].

In our study, we found that maternal breastfeeding *intent* at time of delivery was associated with increased risk of food allergies. Given the retrospective nature of our data collection and since we did not contact mothers directly, we do not know the extent to which maternal intent to breastfeed at time of delivery translated into initiation/duration of breastfeeding. With 79% of mothers in our cohort indicating initial intent to breastfeed, the observed correlation between breastfeeding and food allergies in our data may be attributed to reverse causation, as has been reported previously, since breastfeeding intent and exclusive breastfeeding after hospital discharge may differ considerably [56-59]. Given the limitations imposed by retrospective data collection regarding breast-feeding decision and duration, we cannot draw definitive conclusions about its role in food-allergy development in our cohort.

Although we did not find an association between food allergies and type of delivery, maternal intrapartum receipt of antibiotics, or NICU placement, these relationships warrant further study. Technical limitations were imposed by our dependence upon the ICD-9-CM coding system to identify children with food allergies, as this system is an imperfect surrogate for allergy diagnosis. As a result, we were not able to identify the number of food allergic children that we had initially set out to include in our dataset. When initially established, ICD-9-CM codes were created for insurance reimbursement purposes, not for research purposes. Furthermore, there may be tendencies, on the part of clinicians, to assign specific ICD-9-CM codes to a particular group of signs and symptoms based on reimbursement levels. This may explain why we initially identified 235 children with food allergy-diagnosis-codes born at our institution, but whose diagnosis was only substantiated (by IgE or SPT) in 85 of them. Indeed, we observed that food allergy specific ICD-9-CM codes were used for parent-reported food intolerances, as well as allergies to non-food items (e.g., latex allergy). Recently, Clark et al. also observed that exclusive reliance upon food-allergy-specific ICD-9-CM codes to identify patients with food allergies, would have led to a ~50% discrepancy from the true number of patients with food hypersensitivities [60].

However, a particular strength of our data set is the rigor with which we identified children as “cases” only if an IgE-mediated food allergic reaction had been confirmed [61,62]. By focusing exclusively on children with IgE-mediated food allergies, we may have failed to identify children with food allergic manifestations that are mediated by non-IgE mechanisms [63,64]. Rather than relying upon retrospective review of medical and billing records, future investigations into the role of perinatal and immunological factors impacting food allergies should be initiated prospectively at birth.

## Conclusion

In conclusion, in our cohort of children with IgE-mediated food allergies, we found that males are at a greater risk of developing food allergies than females, and older maternal age at time of delivery is associated with an increased risk of food allergy diagnosis in offspring. These demographic associations could be associated with disruptions in acquisition of gut flora and are worthy of further exploration. It is conceivable that male neonates born to older mothers might benefit the most from early intervention with probiotic therapies, but this remains to be explored.

## Abbreviations

GALT: Gut associated lymph tissue; PBMC: Peripheral blood mononuclear cells; PSHMC: Penn State Hershey Medical Center.

## Competing interests

The authors declare they have no competing interests.

## Authors’ contributions

KK conceived of the project; IP and TF participated in study design; JAL and SS carried out co-incubation assays; NS assisted with technical aspects of cytokine analysis; KEV helped to draft the manuscript; DM provided statistical analysis; JP performed data abstraction. All authors read and approved the manuscript.

## Authors’ information

KK is an Associate Professor in the Department of Pharmacology, where she serves as director of pharmacology medical education.

IP is a Professor in the Departments of Pediatrics and Public Health Sciences.

JAL and SS are medical students.

NS is a research associate in the Department of Pharmacology.

KEV is a Professor and Chairman of the Department of Pharmacology.

DM is a Professor of Public Health Sciences and Division Chief of Biostatistics and Bioinformatics.

TF is an Assistant Professor of Pediatrics and Section Chief of Allergy and Immunology.

JP is a Research Project Manager for the Department of Medicine.

## Supplementary Material

Additional file 1 **Figure S1. ***In vitro* Coincubation. De-identified PBMCs (2 × 10^6^ cells/mL) were collected from 6 healthy adult donors and 6 cord blood donors (IRB oversight #36219NHR and #33295NHR) and co-incubated with lactobacilli and bifidobacteria for 24 hours according to the method described by Foligne et al (reference below). Significant increases in mean levels of (A) IFN-γ (p=0.004), (C) IL-10 (p=0.001), and (E) IL-12 (p=0.001) and were observed following co-incubation of adult-derived cells. Cord blood-derived PBMCs exposed to the bacterial cocktail nearly doubled (B) IFN-γ secretion (p=0.03), (D) increased IL-10 (p=0.001) secretion by 130-fold, (F) but did not lead to substantial changes in IL-12 secretion (2.69 ±2.35 pg/mL to 2.85 ±1.91 pg/mL; p=0.40). Foligne B, Nutten S, Grangette C, Dennin V, Goudercourt D, Poiret S, Dewulf J, Brassert D, Mercenier A, Pot B: **Correlation between*in vitro*and*in vivo*immunomodulatory properties of lactic acid bacteria.***World J Gastroenterol* 2007, **13**:236-243. (DOC 706 kb) Click here for file
