# Supplementary material for: A retrospective chart review to identify perinatal factors associated with food allergies
Source: Nutr J. 2012 Oct 19;11:87. doi: 10.1186/1475-2891-11-87 (PMC3493351; doi:10.1186/1475-2891-11-87)
Supplement: Additional file 1 — Figure S1. In vitro Coincubation. De-identified PBMCs (2 × 10^6^ cells/mL) were collected from 6 healthy adult donors and 6 cord blood donors (IRB oversight #36219NHR and #33295NHR) and co-incubated with lactobacilli and bifidobacteria for 24 hours according to the method described by Foligne et al (reference below). Significant increases in mean levels of (A) IFN-γ (p=0.004), (C) IL-10 (p=0.001), and (E) IL-12 (p=0.001) and were observed following co-incubation of adult-derived cells. Cord blood-derived PBMCs exposed to the bacterial cocktail nearly doubled (B) IFN-γ secretion (p=0.03), (D) increased IL-10 (p=0.001) secretion by 130-fold, (F) but did not lead to substantial changes in IL-12 secretion (2.69 ±2.35 pg/mL to 2.85 ±1.91 pg/mL; p=0.40). Foligne B, Nutten S, Grangette C, Dennin V, Goudercourt D, Poiret S, Dewulf J, Brassert D, Mercenier A, Pot B: Correlation betweenin vitroandin vivoimmunomodulatory properties of lactic acid bacteria.World J Gastroenterol 2007, 13:236-243. (DOC 706 kb) [file 1475-2891-11-87-S1.doc]

Additional Materials

Additional Materials Legend.

*In vitro* Coincubation

De-identified PBMCs (2 × 10^6^ cells/mL) were collected from 6 healthy adult donors and 6 cord blood donors (IRB oversight #36219NHR and #33295NHR) and co-incubated with lactobacilli and bifidobacteria for 24 hours according to the method described by Foligne et al (reference below). Significant increases in mean levels of (A) IFN-γ (p=0.004), (C) IL-10 (p=0.001), and (E) IL-12 (p=0.001) and were observed following co-incubation of adult-derived cells. Cord blood-derived PBMCs exposed to the bacterial cocktail nearly doubled (B) IFN-γ secretion (p=0.03), (D) increased IL-10 (p=0.001) secretion by 130-fold, (F) but did not lead to substantial changes in IL-12 secretion (2.69 ±2.35 pg/mL to 2.85 ±1.91 pg/mL; p=0.40).

Folignfce B, Nutten S, Grangette C, Dennin V, Goudercourt D, Poiret S, Dewulf J, Brassert D, Mercenier A, Pot B: **Correlation between *in vitro* and *in vivo* immunomodulatory properties of lactic acid bacteria.** *World J Gastroenterol* 2007, **13**:236-243.
